# New concept for the prevention and treatment of metastatic lymph nodes using chemotherapy administered via the lymphatic network

**DOI:** 10.1038/srep32506

**Published:** 2016-09-01

**Authors:** Tetsuya Kodama, Daisuke Matsuki, Asuka Tada, Kazu Takeda, Shiro Mori

**Affiliations:** 1 Laboratory of Biomedical Engineering for Cancer, Biomedical Engineering Cancer Research Center, Graduate School of Biomedical Engineering, Tohoku University, 4-1 Seiryo, Aoba, Sendai, Miyagi, 980-8575, Japan; 2 Graduate School of Engineering, Tohoku University, 6-6-05 Aramaki-aza-Aoba, Aoba, Sendai 980-8579, Japan; 3 Department of Oral and Maxillofacial Surgery, Tohoku University Hospital, 1-1 Seiryo, Aoba, Sendai 980-8575, Japan

## Abstract

Intravenous chemotherapy has poor access to metastatic lymph nodes (LNs) and is limited by short-lived drug concentrations. Here, we describe the administration of chemotherapy via the lymphatic network as a new concept for the prevention and treatment of metastatic LNs. A metastatic LN can be treated by the injection of drugs into an upstream LN, either the sentinel LN (SLN) or another upstream LN. In a mouse model, tumor cells were inoculated into the subiliac LN (SiLN) to induce metastasis to the proper axillary LN (PALN). Two routes were used for drug delivery to the PALN, namely from the SiLN and from the accessory axillary LN (AALN). We found that tumor masses were formed in lymphatic vessels between the SiLN and PALN. The flow of fluorescent solution injected into the SiLN towards the PALN decreased with tumor mass formation. Delivery from the AALN (free of metastatic tumor cells) to the PALN was identified as an alternative route. Intranodal injection can deliver high concentrations of drugs to secondary metastatic LNs. The study advocates a new concept for the prevention and treatment of metastatic lymph nodes whereby drugs injected into upstream lymph nodes can reach metastatic lymph nodes via the lymphatic network.

Cancer is now the leading cause of death, and mortality in about 90% of all patients with cancer is attributed to metastasis^1^. Epithelial cancer cells commonly metastasize to draining lymph nodes (LNs) via lymphatic vessels, and the first few LNs into which tumor cells drain are called sentinel LNs (SLNs). In patients, the SLN may be described as either negative (cancer not detected in the LN) or positive (cancer detected in the LN) for metastasis. Axillary LN dissection (ALND) is frequently employed in the treatment of breast cancer with lymphatic metastasis. However, the anatomic disruption caused by ALND can lead to adverse effects such as lymphedema and nerve injury. SLN biopsy was developed to reduce these adverse effects and has emerged as a potential alternative to routine ALND in clinically node-negative breast cancer. Additional LNs in the area may also be surgically removed. However, even if the regional axillary LNs are removed, the possibility remains that micrometastasis or occult metastasis has already occurred to non-dissected LNs, in which case it is not possible to treat fully LN metastasis. In the clinic, intravenous chemotherapy is used for the management of micrometastases in LNs^2^. However, intravenous chemotherapy cannot easily reach the metastases because small molecules, such as anti-cancer drugs, in the interstitium are preferentially re-absorbed into blood capillaries; thus, it is difficult to maintain a high concentration of drug at the target site^3^. Therefore, the development of a new, selective and effective chemotherapeutic method to deliver drugs to metastatic tumor cells in LNs is needed.

In the present study, we utilized an animal model of LN metastasis to provide proof of concept for a novel lymphatic drug delivery system that could be used to treat or prevent micrometastasis to LNs^3^,^4^,^5^,^6^,^7^. Drug was injected, before surgical resection, into an upstream LN (considered to be within the dissection area) in order to deliver the drug to a downstream metastatic LN (considered to be outside the dissection area) and maintain a high drug concentration within the downstream LN. Importantly, we examined the potential of the SLN and an alternative upstream LN as suitable sites for drug injection that would deliver drug to the downstream metastatic LN. In our mouse model, the subiliac LN (SiLN) and two axillary LNs, namely the proper axillary LN (PALN) and accessory axillary LN (AALN), were used. There are two lymphatic routes to the subclavian vein (SV) in the axillary area; SiLN → PALN → SV and AALN → PALN → SV^8^. The SiLN was inoculated with tumor cells and defined as the SLN; the PALN was defined as the downstream secondary LN; and the AALN was defined as an upstream LN (relative to the PALN) that was free from metastases (since there are no lymphatic routes from the SiLN to the AALN or from the PALN to the AALN). Drug flow from the SiLN or AALN to the PALN was evaluated by the injection of an imaging dye into the tumor-bearing SiLN or the AALN.

## Results

### Communication between venous and lymphatic systems in the mouse: anatomical considerations

**Figure 1:**
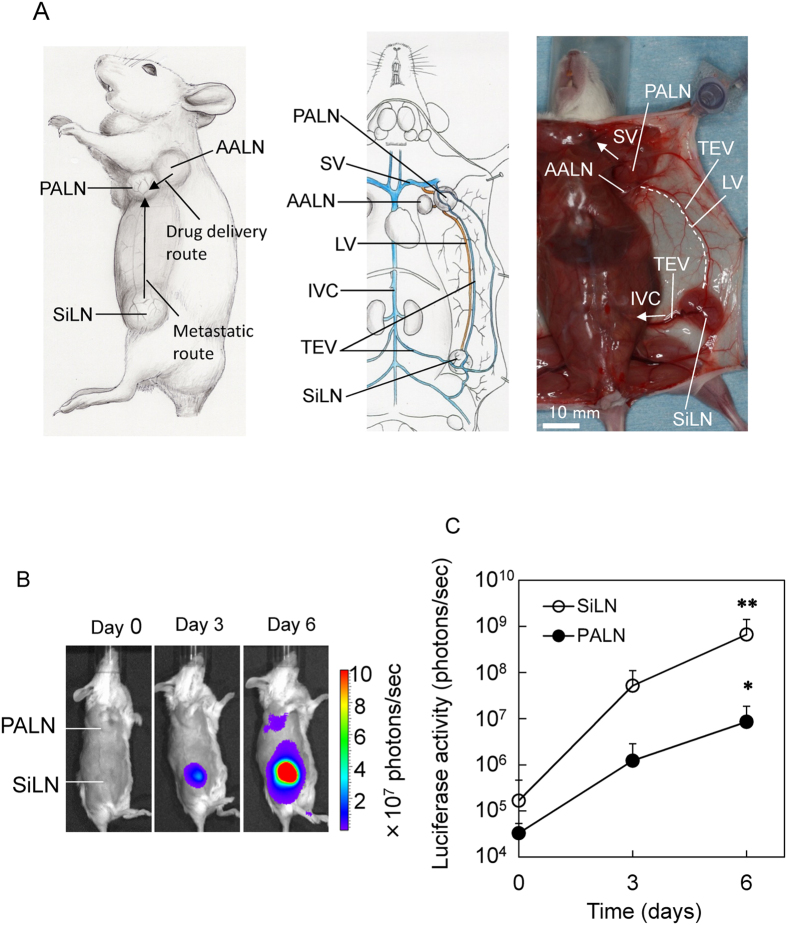
Establishment of a model for lymph node metastasis. (**A**) Gross anatomy in MXH10/Mo/lpr mice. The efferent lymphatic vessels of the SiLN connect to the PALN, and the efferent lymphatic vessels of the PALN connect to the subclavian vein (SV). The efferent lymphatic vessels of the AALN connect to the PALN. Both the SiLN and AALN are located upstream of the PALN. The thoracoepigastric vein (TEV) connects to the SV and inferior vena cava (IVC) via the PALN and SiLN. LV: lymphatic vessel. Arrow: SiLN → PALN (metastatic route); AALN → PALN (lymphatic drug delivery route). Scale bar: 10 mm. (**B**) Induction of metastasis in the PALN. Tumor cells were inoculated into the SiLN to induce metastasis in the PALN. Metastasis to the PALN was detected on day 6 after inoculation. (**C**) Luciferase activity in the SiLN and PALN (*n* = 25). **P < 0.01, day 0 *vs* day 6 in the SiLN; *P < 0.05, day 0 *vs* day 6 in the PALN; one-way ANOVA. Mean ± SD.

First, we describe the network between the venous and lymphatic systems in MXH10/Mo-*lpr*/*lpr* (MXH10/Mo/lpr) mice^8^ (Fig. 1A). The lymphatic system and venous system were identified in the axillary and subiliac regions. In the axillary area, there are two LNs; one is the PALN, the other the AALN^9^. The efferent lymphatic vessels of the AALN connect to the PALN^6^. The efferent lymphatic vessels of the SiLN connect to the PALN, while the efferent lymphatic vessels of the PALN connect to the subclavian vein. The thoracoepigastric vein (TEV), into which blood drains from the PALN and SiLN, connects to the subclavian vein (SV) and the inferior vena cava (IVC)^8^.

### LN metastasis from the SiLN to the PALN

Injection of tumor cells into the SiLN induced metastasis in the PALN (Fig. 1B). Tumor progression in the SiLN and PALN was measured using an *in vivo* bioluminescence imaging system. Metastasis in the PALN was detected on day 6 after inoculation of tumor cells into the SiLN (**P < 0.01, day 0 *vs* day 6 in the SiLN; *P < 0.05, day 0 *vs* day 6 in the PALN) (Fig. 1C).

### Delivery rate to the PALN after the injection of fluorescent solution into the SiLN

**Figure 2:**
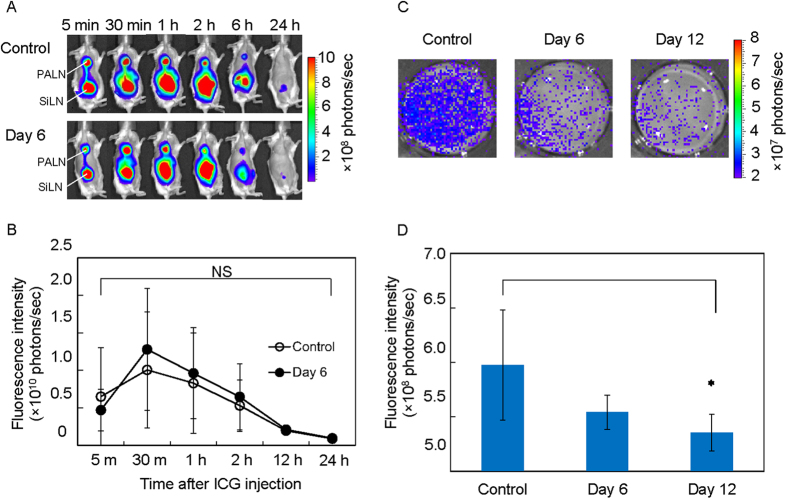
Fluorescence intensity of ICG in the PALN. (**A**) Representative images of mice after the injection of ICG solution into the SiLN. (**B**) Changes in the fluorescence intensity of ICG solution in the PALN over time. NS: not significant. Control (inoculation of vehicle without tumor cells), *n* = 4; day 6 (after inoculation of tumor cells), *n* = 7. (**C**,**D**) The PALN was homogenized and the fluorescence intensity of the supernatant was measured by IVIS. (**C**) Fluorescence images of individual wells of a 12-well plate. (**D**) Averaged values for the fluorescence intensity in the control group (inoculation of vehicle without tumor cells) and on day 6 and day 12 after inoculation of tumor cells (control, *n* = 4; day 6, *n* = 4; day 12, *n* = 4). The mean ± SD values are shown. *P < 0.05, control *vs* day 12 (Mann-Whitney U-test).

It was determined whether the SLN could be used as an injection site to deliver drugs to the secondary metastatic LN (Fig. 2A,B). Indocyanine green (ICG) solution was injected into the tumor-bearing SiLN (the SLN) on day 6 after tumor cell inoculation, and time-dependent changes in fluorescence intensity in the PALN were measured (Fig. 2A,B). When ICG solution was injected into the SiLN, the solution flowed into the efferent lymphatic vessels and the TEV^6^,^8^. The fluorescence intensity in the PALN reached a maximum value 30 min after injection for both the control (inoculation of vehicle without tumor cells) and day 6 (inoculation of tumor cells) groups, and subsequently decreased to the background level (1 × 10^9^ photons/sec) after 24 h. There was no significant difference between the control and tumor groups with regard to the time-dependent changes of fluorescence intensity in the PALN. When ICG solution was injected into the SiLN, it also flowed into the TEV and the efferent lymphatic vessel^10^. Although ICG solution tended to accumulate in organs of the reticuloendothelial system such as the liver, spleen and lungs, which it reached via the blood circulation^11^, its accumulation in these organs was not measured in the present experiments.

### *Ex vivo* evaluation of the delivery rate to the PALN after injection of fluorescent solution into the SiLN

Next, we harvested the PALN and measured the delivery of ICG solution to the PALN 30 min after injection of ICG solution into the SiLN (Fig. 2C,D). ICG fluorescence in the PALN was observed in all groups (control, day 6 and day 12). ICG fluorescence intensity was significantly lower on day 12 after tumor cell inoculation than in the control group (*P < 0.05, control *vs* day 12).

### Morphological changes in lymphatic vessels during the progression of metastasis

**Figure 3:**
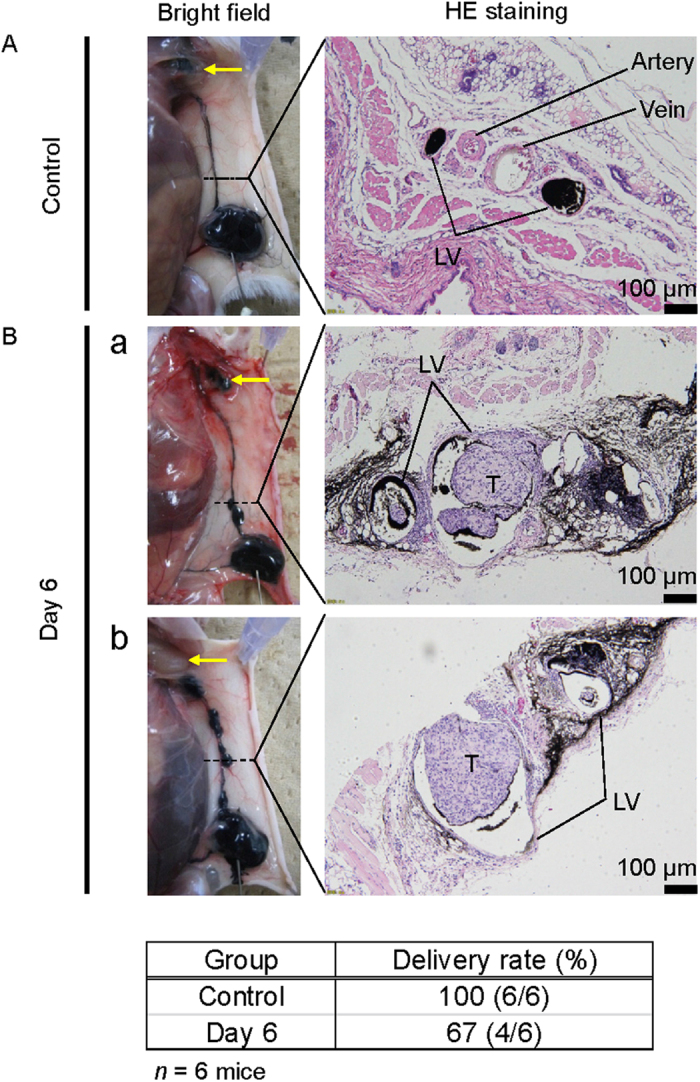
India ink flow from the SiLN to the PALN and histopathological analysis. (**A**) Control group (*n* = 6). India ink was delivered to the PALN in all animals (delivery rate: 100%). Histopathology (H&E) revealed an artery and vein located between two lymphatic vessels stained with India ink. (**B**) Day 6 group (*n* = 6). (a) India ink was delivered from the SiLN to the PALN in 4/6 animals (delivery rate: 67%). (b) India ink was not delivered to the PALN in 2/6 animals (33%). The lymphatic vessels between the SiLN and PALN were harvested 30 min after the injection of India ink. Tissue sections stained with H&E demonstrated lymphatic vessels filled with tumor cells. Yellow arrow: PALN; LV: lymphatic vessel; T: tumor cells. The table indicates the rate of India ink delivery from the SiLN to the PALN.

The ICG fluorescence intensity in the PALN was observed to decrease as the time after inoculation of cells into the SiLN increased (Fig. 2). To investigate further flow from the SiLN to the PALN after tumor cell inoculation, India ink (100 μL) was injected into the SiLN on day 6. The lymphatic vessels between the SiLN and PALN were harvested 30 min after the injection of India ink and stained with hematoxylin and eosin (H&E). In controls (Fig. 3A), the India ink injected into the SiLN was delivered to the PALN in 6/6 animals (a rate of 100%), resulting in the PALN being stained black (arrow, Fig. 3A). In tissue sections stained with H&E, two lymphatic vessels running between the SiLN and PALN were stained black with India ink; a vein and an artery were located between the two lymphatic vessels (Fig. 3A). When similar experiments were carried out on day 6 after tumor cell inoculation (Fig. 3B), India ink was delivered to the PALN in only 4/6 animals (67%) (Fig. 3Ba). Furthermore, animals in which delivery of India ink to the PALN failed (Fig. 3Bb) were found to have more tumor masses in the lymphatic vessels than animals in which delivery to the PALN succeeded.

### Visualization of flow from the SiLN and AALN to the PALN

**Figure 4:**
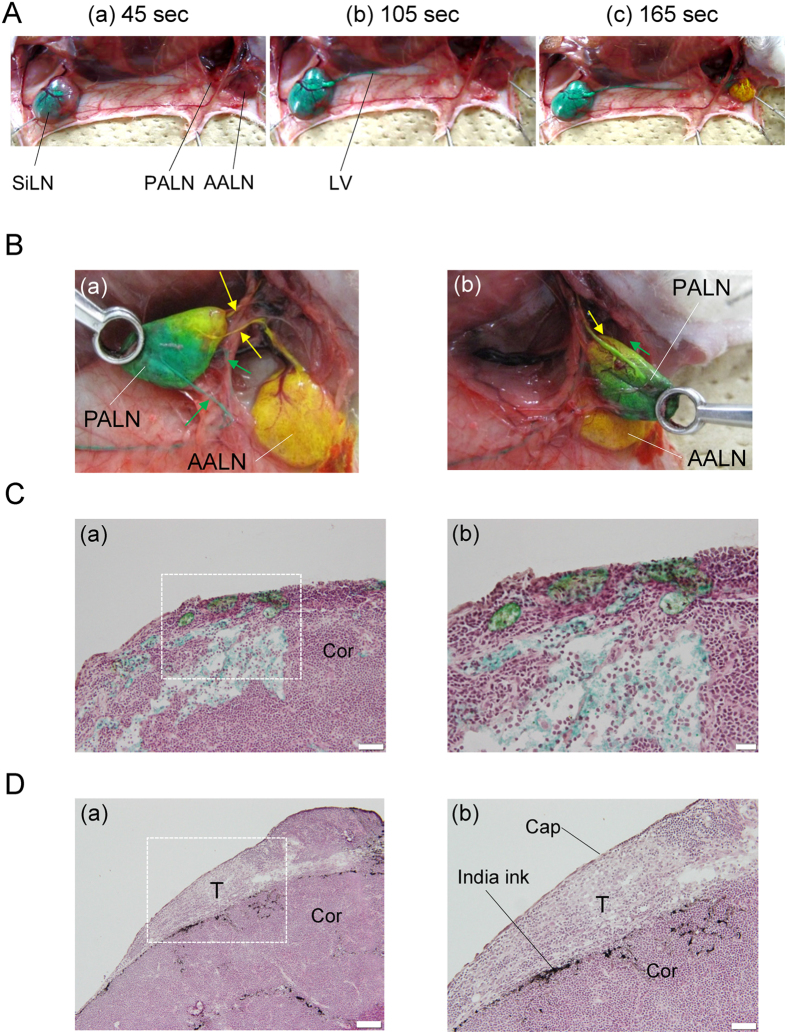
Lymphatic delivery from the SiLN and AALN to the PALN. (**A**) Visualization of two lymphatic routes. Green dye was driven to flow from the SiLN towards the PALN at a velocity of 50 μL/min using a syringe pump. At 120 sec after the initiation of green dye injection, yellow dye was driven from the AALN to the PALN at a velocity of 50 μL/min using a second syringe pump. Both administrations were terminated 180 sec after the initiation of green dye injection. The total volume of green dye injected was 150 μL, while that of yellow dye was 50 μL. (a) 45 sec after starting the injection of green dye. Approximately half of the SiLN was filled with green dye. (b) 105 sec after starting the injection of green dye. Green dye was visualized as running in the efferent lymphatic vessels of the SiLN towards the PALN. (c) 165 sec after starting the injection of green dye. Green and yellow dyes had converged in the PALN. (**B**) PALN showing the convergence of green and yellow dyes. (a) Anterior aspect. Two efferent lymphatic vessels extended from the SiLN to the PALN and two efferent lymphatic vessels extended from the AALN to the PALN. The area where the two dyes converged was dyed greenish-yellow. (b) Posterior aspect. Some of the green dye had flowed into the afferent lymphatic vessel of the PALN. (**C**) The marginal sinus of the PALN was filled with green and yellow dye (*n* = 3). (a) The dyes had spread into the cortex. (b) The square region in (a). H&E staining. Cor: cortex. Scale bar: (a) 100 μm, (b) 20 μm. (**D**) Metastatic tumor cells formed in the marginal sinus of the PALN on day 9 after inoculation of cells into the SiLN (*n* = 3). Tumor (T) was detected in the marginal sinus of the PALN. H&E staining. (a) India ink was delivered into the marginal sinus of the PALN. (b) The square region in (a). India ink was delivered below the tumor. Cap: capsule; Cor: cortex. Scale bar: (a) 100 μm, (b) 50 μm.

In order to visualize two separate lymphatic routes from the SiLN and AALN to the PALN, we injected green and yellow dye into the SiLN and AALN, respectively (Fig. 4A). The green dye was injected into the SiLN over a period of 180 sec; the yellow dye was injected into the AALN over a period of 60 sec, and was started 120 sec after the initiation of green dye injection to ensure that both injections terminated at the same time. At 45 sec after starting the injection, approximately half of the SiLN was filled with green dye (Fig. 4Aa). At 105 sec, the entire SiLN was filled with green dye, and part of the efferent lymphatic vessels of the SiLN also contained green dye (Fig. 4Ab). At 165 sec, part of the PALN contained green dye, while the AALN was filled with yellow dye. Some of the yellow dye was delivered to the PALN (Fig. 4Ac). Figure 4B shows that the two dyes met in the PALN after termination of both injections. Two efferent lymphatic vessels extended from the SiLN to the PALN (green arrows), while two efferent lymphatic vessels extended from the AALN to the PALN (yellow arrows). The area where the two dyes converged was colored greenish-yellow (Fig. 4Ba). Figure 4Bb shows the posterior aspect of the PALN in Fig. 4Ba, demonstrating that some of the green dye had flowed into the afferent lymphatic vessel of the PALN. Histologic analysis revealed that the marginal sinus of the PALN was filled with green and yellow dyes (Fig. 4Ca,b), and that the dyes had spread into the cortical regions.

Next, we investigated whether an upstream LN that was free from metastasis might be a suitable injection site to deliver drugs to a secondary metastatic LN. We induced metastasis in the PALN by inoculating tumor cells into the SiLN, and injected India ink into the AALN on day 9 after inoculation (Fig. 4D). Tumor formed in the marginal sinus of the PALN. India ink was delivered from the AALN into the marginal sinus of the PALN and spread into the cortex (Fig. 4Da). India ink was delivered below the tumor (Fig. 4Db).

## Discussion

This is the first paper, utilizing an MXH10/Mo/lpr mouse model of lymphatic metastasis that demonstrates the new concept that secondary metastatic LNs could potentially be treated by the injection of drugs into either the SLN or an upstream LN that is free from metastasis. In this model, tumor cells were inoculated into the SiLN to induce metastasis to the PALN. Drug that was injected into the SiLN (i.e., pseudo-SLN) or AALN (free from metastasis) was able to reach tumor cells in the marginal sinus of the PALN via the lymphatic vessels. MXH10/Mo/lpr mice exhibit systemic lymphadenopathy, with some peripheral LNs as large as 10 mm in diameter^9^. The lymphadenopathy in these mice is due to the accumulation of lpr T cells in the LNs^12^,^13^,^14^,^15^. However, the metastasis process that begins in the marginal sinus is universal and may be independent of lpr-induced intranodal structural changes.

Cady *et al*.^16^ have updated the results of Harvey and Auchincloss^17^, investigating 10-year disease-free survival in human breast, colorectal, gastric and lung cancer. In the updated results, it was determined that survivors seldom harbored more than three regional LN metastases from their original cancer. However, it is not clear whether these LNs were connected by lymphatic vessels in long strands or whether tumor cells metastasized directly to these LNs through the lymphatic vessels.

Our data revealed that the flow of fluorescent solution to the secondary metastatic LN, i.e. PALN, decreased during tumor progression and instead was oriented to the systemic circulation via the TEV. This finding indicates that tumor cells do not metastasize in long strands but are diverted from the SLN to the systemic circulation, i.e. the final form of LN metastasis should be recognized as “*LN-mediated hematogenous metastasis*^8^.” Therefore, in order to inhibit systemic metastasis from LNs containing secondary tumor, drugs must be injected into the SLN^3^,^7^ during the early stages of LN metastasis.

Clinical^18^,^19^ and experimental^20^,^21^ studies have reported that a massive metastasis in the SLN may block the lymphatic route from the primary tumor to the SLN, resulting in a rerouting of lymphatic flow from the primary tumor to a neo-SLN. This phenomenon depends on the volume occupied by the tumor mass in the SLN (i.e., tumor progression) and the lymphatic network between the primary tumor and SLN. Thus, the existence of tumor cells in the SLN does not necessarily lead to a rerouting of lymphatic flow from the primary tumor to a neo-sentinel lymph node.

The method proposed in this study could potentially maintain a high drug concentration in both the SLN and the secondary metastatic LNs. Drugs would be able to access secondary metastatic LNs from the SLN until flow through the connecting lymphatic vessels ceased; at this point, an upstream LN other than the SLN would need to be selected as an alternative drug injection site to deliver drugs to the secondary metastatic LNs. Applying our concept to clinical practice, the detection of even the early stages of LN metastasis by the imaging system used here would raise the possibility that the lymphatic network is already blocked by invading tumor cells. Therefore, the novel therapy we propose should be directed at LNs that are clinically negative for metastasis. Since it is difficult to visualize the lymphatic network during surgery, there is also the possibility for the lymphatic network to be damaged by the surgical procedure. To overcome these issues, ICG (or radioisotope) could be injected into the vicinity of the primary tumor to identify the SLNs. Then, under the guidance of ultrasound imaging, anti-cancer agents could be injected into the SLNs. Once the anti-cancer agents had been delivered to the secondary LNs via the lymphatic routes from the SLNs, the SLNs could be resected for biopsy if required. In this way, the secondary LNs would receive potentially curative or preventative treatment even if the SLNs were subsequently removed.

Direct injection of drugs into the LNs has been established using techniques such as ultrasound-guided internal jugular vein catheterization. Therefore, the present method has the potential to treat and/or prevent LN metastasis at the earliest stage in the clinical setting. Prospective studies are merited to further examine and validate the concept proposed in this study.

## Materials and Methods

Experiments were carried out in accordance with approved guidelines, and were approved by the Institutional Animal Care and Use Committee of Tohoku University.

### Mice

MXH10/Mo-*lpr*/*lpr* (MXH10/Mo/lpr) mice (16–18 weeks) were bred under specific pathogen-free conditions in the Animal Research Institute, Tohoku University. MXH10/Mo/lpr mice are unique; their most peripheral LNs grow to 10 mm in size at 2.5–3 months of age, and notably the mice do not develop severe autoimmune diseases^9^.

### Cell culture

Malignant fibrous histiocytoma-like KM-Luc/GFP cells, stably expressing a fusion of luciferase and enhanced-green fluorescent protein genes, were used^22^. Cells were cultivated in Dulbecco’s modified Eagle’s medium (DMEM; Sigma-Aldrich, St Louis, MO, USA) supplemented with 10% heat-inactivated fetal bovine serum and 1% L-glutamine–penicillin–streptomycin (Sigma-Aldrich). Cells were incubated (37 °C, 5% CO_2_/95% air) until 80% confluence was achieved. Lack of *Mycoplasma* contamination was confirmed on the inoculation day (MycoAlert *Mycoplasma* Detection Kit; Lonza Rockland, Allendale, NJ, USA).

### Induction of metastasis to the PALN by injection of tumor cells into the SiLN

Metastasis to the PALN was induced by injecting 3.3 × 10^5^ cells suspended in a mixture of 20 μL phosphate-buffered saline (PBS) and 40 μL of 400 mg/mL Matrigel (Collaborative Biomedical Products, Bedford, Canada) into the unilateral SiLN (*n* = 25). In control animals, 60 μL of vehicle (20 μL PBS plus 40 μL Matrigel) without cells was injected (*n* = 14). Inoculation was carried out using a 24-gauge needle under the guidance of a high-frequency US imaging system (VEVO770; VisualSonics, Toronto, Canada) with a 25-MHz transducer (RMV-710B; axial resolution, 70 μm; focal length, 15 mm; VisualSonics). Each mouse was anesthetized with 2% isoflurane in oxygen, and the day of inoculation was defined as day 0.

### Detection of tumor growth and metastasis by *in vivo* bioluminescence imaging

Metastasis to the PALN was assessed using an *in vivo* bioluminescence imaging system (IVIS; Xenogen, Alameda, CA, USA) on days 0, 3 and 6 for KM-Luc/GFP cells. The background luciferase activity in controls was ~4 × 10^4^ photons/sec. Mice whose luciferase activity in the PALN was larger than the background luciferase activity were considered as being metastatic mice. Each mouse was anesthetized with 2.0% isoflurane in oxygen, and 150 mg/kg luciferin (Promega, Madison, WI, USA) was injected intraperitoneally. After 10 min, luciferase bioluminescence was measured for 30 sec, using IVIS. The metastatic mice were divided into two groups: a day 6 group consisting of mice examined 6 days after inoculation of tumor cells (*n* = 17) and a day 12 group consisting of mice examined 12 days after inoculation of tumor cells (*n* = 4).

### Delivery rate to the PALN after injection of fluorescent solution into the SiLN

40 μL of 62.5 μg/mL ICG solution (Daiichi Sankyo, Tokyo, Japan) was injected at a velocity of 50 μL/min, using a syringe pump, into the unilateral SiLN of mice on day 6 (*n* = 7) and the control (*n* = 4) groups^22^. The fluorescence intensity of the PALN was assessed using an *in vivo* bioluminescence imaging system (IVIS; Xenogen). This procedure was carried out at 5 min, 30 min, 1 h, 2 h, 6 h and 24 h after the injection of ICG solution.

### *Ex vivo* evaluation of delivery rate to the PALN after injection of fluorescent solution into the SiLN

40 μL of 62.5 μg/mL ICG solution was injected at a velocity of 50 μL/min, using a syringe pump, into the unilateral SiLN of mice on day 6 (*n* = 4), day 12 (*n* = 4) and the control (*n* = 4) groups. The PALN was dissected and homogenized, and the fluorescence intensity of the tissue suspension was assessed using an *in vivo* bioluminescence imaging system (IVIS; Xenogen).

### Flow of India ink from the SiLN to the PALN during the progression of metastasis

100 μL of black India ink was injected, using a syringe pump, at a velocity of 50 μL/min into the unilateral SiLN of mice on day 6 (*n* = 6) and the control (*n* = 6) groups.

### Visualization of the lymphatic routes from the SiLN and AALN to the PALN

Under anesthesia, an arc-shaped incision was made in the abdominal skin from the subiliac to the proper axillary region. Green and yellow dyes (Pentel, Tokyo, Japan) were injected into the SiLN and AALN, respectively (*n* = 3). Both dyes were dissolved in PBS (dye: PBS = 2:1). The two dyes were injected independently using separate syringes, 27G butterfly needles and syringe pumps. The green dye was injected into the center of the SiLN and the yellow dye into the center of the AALN. First, green dye was driven to flow from the SiLN towards the PALN at a velocity of 50 μL/min using a syringe pump. At 2 min after the initiation of green dye injection, yellow dye was driven from the AALN to the PALN at a velocity of 50 μL/min using another syringe pump. Both administrations were terminated 3 min after the start of the first injection. The total volume of green dye injected was 150 μL and that of yellow dye was 50 μL. Subsequently, the mouse was sacrificed, and the PALN was harvested, mounted in optimal cutting temperature (OCT) compound, frozen and stored at −80 °C.

### Delivery of India ink from the AALN to metastatic tumor in the PALN

On day 6 after inoculation of tumor cells into the SiLN, India ink was injected into the AALN for 60 sec at a velocity of 50 μL/min using a syringe pump (*n* = 4). The mouse was then sacrificed, and the PALN was harvested, mounted in OCT compound, frozen and stored at −80 °C.

### Histological analysis

Paraffin-embedded specimens were cut into 2-μm serial sections. Frozen samples were cut into 10-μm sections using a cryostat. All sections were stained with H&E.

### Statistical analysis

All measurements are presented as the mean ± SD. Differences between groups were determined by one-way ANOVA (Fig. 1C) followed by the Mann-Whitney U test (Fig. 2B,D). A *P* value < 0.05 was considered to represent a statistically significant result. Statistical analyses were conducted using Excel 2010 (Microsoft) with Statcel2 software.

## Additional Information

**How to cite this article**: Kodama, T. *et al*. New concept for the prevention and treatment of metastatic lymph nodes using chemotherapy administered via the lymphatic network. *Sci. Rep.* **6**, 32506; doi: 10.1038/srep32506 (2016).
